# Serum lipid profiles are associated with disability and MRI outcomes in multiple sclerosis

**DOI:** 10.1186/1742-2094-8-127

**Published:** 2011-10-04

**Authors:** Bianca Weinstock-Guttman, Robert Zivadinov, Naeem Mahfooz, Ellen Carl, Allison Drake, Jaclyn Schneider, Barbara Teter, Sara Hussein, Bijal Mehta, Marc Weiskopf, Jacqueline Durfee, Niels Bergsland, Murali Ramanathan

**Affiliations:** 1 Department of Neurology, State University of New York, Buffalo, NY, USA; 2 Bufalo Neuroimaging Analysis Center, Department of Neurology, State University of New York, Buffalo, NY, USA; 3 Department of Pharmaceutical Sciences, State University of New York, Buffalo, NY, USA

**Keywords:** Multiple sclerosis, diet, lipid profile, MRI, environmental factors, gene-environment interactions, lesion volume, brain atrophy

## Abstract

**Background:**

The breakdown of the blood-brain-barrier vascular endothelium is critical for entry of immune cells into the MS brain. Vascular co-morbidities are associated with increased risk of progression. Dyslipidemia, elevated LDL and reduced HDL may increase progression by activating inflammatory processes at the vascular endothelium.

**Objective:**

To assess the associations of serum lipid profile variables (triglycerides, high and low density lipoproteins (HDL, LDL) and total cholesterol) with disability and MRI measures in multiple sclerosis (MS).

**Methods:**

This study included 492 MS patients (age: 47.1 ± 10.8 years; disease duration: 12.8 ± 10.1 years) with baseline and follow-up Expanded Disability Status Score (EDSS) assessments after a mean period of 2.2 ± 1.0 years. The associations of baseline lipid profile variables with disability changes were assessed. Quantitative MRI findings at baseline were available for 210 patients.

**Results:**

EDSS worsening was associated with higher baseline LDL (*p *= 0.006) and total cholesterol (*p *= 0.001, 0.008) levels, with trends for higher triglyceride (*p *= 0.025); HDL was not associated. A similar pattern was found for MSSS worsening. Higher HDL levels (*p *< 0.001) were associated with lower contrast-enhancing lesion volume. Higher total cholesterol was associated with a trend for lower brain parenchymal fraction (*p *= 0.033).

**Conclusions:**

Serum lipid profile has modest effects on disease progression in MS. Worsening disability is associated with higher levels of LDL, total cholesterol and triglycerides. Higher HDL is associated with lower levels of acute inflammatory activity.

## Introduction and Background

Multiple sclerosis (MS) is a complex inflammatory, demyelinating and neurodegenerative disease with a heterogeneous pathology and clinical outcomes [1]. The chronic inflammatory processes that characterize MS pathology interfere with immune mechanisms that regulate and confine the inflammatory cascade to prevent irreversible tissue damage [2].

Cholesterol is an important component of intact myelin. Lipids, especially lipoproteins, are involved in the regulation of neural functions in the central nervous system through local mechanisms that are linked to systemic lipid metabolism [3,4]. High-density lipoproteins (HDL) and low-density lipoproteins (LDL) play a key role in the transport of cholesterol and lipids in human plasma. Under normal physiological conditions, high concentrations of HDL and LDL are present in CNS as a result of transport across the blood-brain barrier [5,6]. Apolipoprotein A-I, a major component of plasma HDL, is synthesized within the vascular endothelial cells [7]. HDL has immunomodulatory and anti-oxidant effects on endothelial cells [8] and it has been shown to inhibit production of the pro-inflammatory cytokines interleukin-1beta and tumor necrosis factor [9,10]. Apolipoprotein A-1 and paraoxonase are associated with HDL and contribute to its anti-oxidant and anti-inflammatory properties [9,11,12].

Dyslipidemia can potentiate inflammatory processes at the vascular endothelium, lead to the induction of adhesion molecules, and the recruitment of monocytes [13-15]. Associations between dyslipidemia and increased inflammation are well established in conditions such atherosclerosis, cardiovascular disease, metabolic syndrome and obesity [16].

In the context of autoimmune diseases, a strong association between dyslipidemia and cardiovascular disease has emerged in systematic lupus erythematosus [17] and increased cardiovascular risk and lipid profile changes have been reported in rheumatoid arthritis [18]. HDL and LDL also modulate the function and survival of β-cells in Type 2 diabetes mellitus [19]. Neuromyelitis optica patients were reported to have significantly higher serum cholesterol triglycerides and lower LDL than healthy controls [20].

However, only limited information is available on the effect of serum triglycerides and cholesterol levels and the roles of HDL and LDL levels on MS disease progression. Increased total cholesterol was associated with increases in the number of contrast-enhancing lesions on brain MRI in clinically isolated syndrome patients following a first clinical demyelinating event [21]. MS patients were found to have a higher occurrence of hypercholesterolemia and paraoxonase-1, the anti-oxidant enzyme associated with HDL, was decreased during relapses [12]. A retrospective analysis of a large dataset of 8,983 patients from the North American Research Committee on Multiple Sclerosis Registry reported that the presence of vascular comorbidities linked to dyslipidemia was associated with an increased risk for disability progression in MS [22].

The aim of this study therefore was to assess the associations of serum lipid profile variables (serum cholesterol, HDL, LDL and triglycerides) to clinical disability and brain tissue integrity as measured with quantitative magnetic resonance imaging (MRI) metrics in a large cohort of MS patients.

## Methods

### Study Population

#### Ethics Statement

The study was approved by the University at Buffalo Human Subjects Institutional Review Board. The Institutional Review Board approval waived the requirement for informed consent.

#### Study Design

Single-center, retrospective, longitudinal study.

#### Study Population

The study population included consecutive patients, followed at the Baird MS Center, State University of New York, Buffalo, NY, with clinically definite MS patients according to the McDonald criteria [23] with available baseline EDSS assessment within ± 6 months of lipid profile testing and a follow-up EDSS assessment ≥ 6 months from the baseline clinical visit. Patients with CIS and neuromyelitis optica were not included.

The collected data included demographic and clinical information, statin use history, height and weight and fasting lipid profile laboratory values: HDL, LDL, triglycerides, total cholesterol and cholesterol to HDL ratio.

The exclusion criteria consisted of: any relapse with corticosteroid treatment at the time or within one month preceding study entry or MRI examination, pre-existing medical conditions known to be associated with brain pathology (e.g., neurodegenerative disorders, cerebrovascular disease, positive history of alcohol abuse, etc.), and insufficient quality of the MRI scan for quantitative analysis [24].

### MRI Analysis

Quantitative MRI analysis obtained within ± 3 months from the baseline clinical visit (yielding EDSS and fasting cholesterol levels) was available for 210 of 492 patients at baseline. MRI image analysis was conducted at the Buffalo Neuroimaging Analysis Center using approaches previously described [25,26]. MRI analysts were blinded to lipid profile and clinical status. The standardized acquisition and analysis methods for obtaining contrast-enhancing lesion volume (CE-LV), CE lesion number (CEL number), T2-LV, T1-LV and brain parenchymal fraction (BPF) are detailed in Additional File 1.

### Data Analysis

SPSS (SPSS Inc., Chicago, IL, version 15.0) statistical program was used for all statistical analyses.

One-way ANOVA followed by post-hoc independent sample *t*-tests were used to test for differences in means of continuous demographic variables such as age, age of onset, and disease duration. The ^2 ^test was used for analysis of count variables for categorical data and the Fisher exact test was used where appropriate.

The MS Severity Scale (MSSS) was calculated from the EDSS and disease duration values using software downloaded from http://www-gene.cimr.cam.ac.uk/MSgenetics/GAMES/MSSS/Readme.html. The global reference data set provided with the software was used for calculations.

The difference between EDSS at follow-up and EDSS at baseline was analyzed as the dependent variable in regression analysis with gender, disease duration at baseline EDSS, EDSS at baseline, time difference between follow-up and baseline EDSS assessments, statin use and a lipid profile variable of interest (either HDL, LDL, triglycerides, total cholesterol or cholesterol to HDL ratio) as predictor variables. The difference between MSSS at follow-up and MSSS at baseline was analyzed in the same manner as the EDSS; however, the MSSS at baseline was included as a predictor in place of EDSS at baseline and the disease duration was not included as a predictor variable. Similar regression analyses were also conducted in the subset of patients who were not on statins to assess the contributions of lipid profile variables in the absence of statin treatment.

Baseline EDSS was dichotomized into two groups based on EDSS < 4.0 and ≥ 4.0. The baseline EDSS groups were analyzed using logistic regression with sex as a factor and disease duration and lipid profile variable of interest.

The CE-LV, T2-LV and T1-LV data were normalized by cube-root transformation to reduce skew. The cube-root-transformed T2-LV and T1-LV values were analyzed as dependent variables using multiple linear regression. The presence/absence of CE lesions (CEL) was analyzed with logistic regression and the CEL number was analyzed with Poisson loglinear regression and the transformed CE-LV values were analyzed with Tweedie regression [27]. All regression MRI analyses included sex, disease duration at time of MRI, statin use, and a lipid profile variable of interest (either HDL, LDL, triglycerides, total cholesterol or cholesterol to HDL ratio) as predictor variables. Regression analyses were also conducted in the subset of patients who were not on statins to assess the contributions of lipid profile variables in the absence of statin treatment.

To correct for the multiple testing involved, a conservative Type I error level of 0.01 was used to assess significance; a trend was assumed if the Type I error level ≤ 0.10.

## Results

### Demographic and Clinical Characteristics

The clinical, demographic and MRI features of the cohort are summarized in Table 1. The frequency of Caucasian-Americans was 422 (85.8%), African-Americans was 28 (5.7%), Hispanics was 5 (1%), Native American 1 (0.2%), and the racial information for 34 (6.9%) patients was missing.

**Table 1:** Demographic and clinical characteristics of the cohort.

Demographic Variables	Value
Females: Males (% Female)	370: 122 (75.2%)
MS course:	
Relapsing-remitting	395 (80.3%)
Secondary progressive	82 (16.6%)
Primary progressive	15 (3.0%)
Age*, years	47.1 ± 10.8
Disease duration*, years	12.8 ± 10.1
Median EDSS* (IQR)	2.50 (2.50)
MSSS	3.79 ± 2.46
Time to follow-up, years	2.2 ± 1.0
Statin usage^§^	109/491 (22.2%)

**Lipid Profile Variables**	

Body mass index, kg/m^2^	27.8 ± 6.5
HDL, mg/dL	55.2 ± 16.6
LDL, mg/dL	116 ± 32.8
Total cholesterol, mg/dL	197 ± 38.1
Triglycerides, mg/dL	133 ± 82.0
Cholesterol to HDL ratio	3.85 ± 1.30

**MRI Characteristics**	

CEL present	29/197 (14.7%)
CEL number	0.52 ± 0.15
CE-LV, cm^3^	0.032 ± 0.13
T2-LV, cm^3^	14.0 ± 14.7
T1-LV, cm^3^	3.1 ± 5.4
BPF	0.856 ± 0.0285

The continuous variables expressed as mean ± SD and categorical variables as frequency (%).

* At baseline lipid profile assessment. ^§^Statin usage status unavailable for one patient.

The median absolute time difference between lipid profile and baseline EDSS assessment was 25 days (Inter-quartile range: 51 days). The median absolute time difference between MRI and lipid profile assessments was 30 days (Inter-quartile range: 46 days). The median time between baseline EDSS and follow-up EDSS was 1.88 years (Inter-quartile range: 1.62 years).

The majority of patients were on disease-modifying therapies: 45% were on interferon-beta-1a monotherapy, 0.8% were on interferon-beta-1b monotherapy, 14% were on glatiramer acetate, 20% were on natalizumab, 8% were on no therapy and the remainder were on combination therapies or chemotherapies.

MRI data were available for 210 patients. There was no evidence for lipid profile differences between the groups with and without MRI available (See Additional File 1, Table S1). The group with MRI differed from the group without MRI in the higher frequency of progressive forms of MS and a modestly shorter time between baseline EDSS and follow up EDSS (See Additional File 1, Table S1).

The frequency of statin usage was 109/491 patients (22.2%). There was no evidence for differences in the groups with and without statin treatment in the lipid profile variables including HDL, LDL, triglycerides, total cholesterol and cholesterol to HDL. Not surprisingly, the group on statin treatment had a higher proportion of males, greater mean age, disease duration, BMI and baseline EDSS than the group not on statin treatment (Table 2).

**Table 2:** Demographic, clinical and MRI characteristics, and lipid profiles of patient subsets with and without statins.

Variable	No Statins	Statins	*p*-value
Females: Males (% Female)	301: 81 (78.8%)	69: 40 (63.3%)	< 0.002^§^
MS course:			0.075^‡^
Relapsing-remitting	314	81 (74.3%)	
Secondary progressive	(82.2%)		
Primary progressive	55 (12.8%) 13 (3.4%)	27 (24.8%) 1 (0.9%)	
Age*, years	45.3 ± 10.7	53.4 ± 8.4	< 0.001
Disease duration*, years	11.9 ± 9.7	16.2 ± 11.0	< 0.001
Median EDSS* (IQR)	2.5 (2.0)	3.50 (3.50)	< 0.001^#^
MSSS	3.67 ± 2.51	4.15 ± 2.18	0.061
Time to follow-up, years	2.13 ± 1.0	2.22 ± 1.0	0.43

Body mass index, kg/m^2^	27.4 ± 6.5	29.1 ± 6.5	0.013^¶^
HDL, mg/dL	55.6 ± 16.6	53.7 ± 16.5	0.72^¶^
LDL, mg/dL	115 ± 30.8	118 ± 39.0	0.62^¶^
Total cholesterol, mg/dL	196 ± 36.0	201 ± 44.7	0.38^¶^
Triglycerides, mg/dL	128 ± 84.0	149 ± 75.5	0.15^¶^
Cholesterol to HDL ratio	3.81 ± 1.28	4.00 ± 1.40	0.60^¶^

Presence of CEL CEL number CE-LV, cm^3^	25/156 (16%) 0.60 ± 2.3 0.035 ± 0.15	4/40 (10%) 0.23 ± 0.86 0.019 ± 0.080	0.47^¶^ 0.026^¶^ 0.035^¶^
T2-LV, cm^3^	13.6 ± 14.7	16.0 ± 14.7	0.30^¶^
T1-LV, cm^3^	2.7 ± 5.1	4.5 ± 6.3	0.019^¶^
BPF	0.858 ± 0.027	0.848 ± 0.035	0.056^¶^

Statin usage data were available for 491 patients.

* At time of baseline lipid profile assessment.

^§ ^Fisher exact test

^‡ ^Fisher exact test for presence of secondary progressive or progressive forms of MS.

# Mann-Whitney test

^¶ ^*p*-values for statin variable from regression analyses with sex, disease duration and statin use as predictor variables.

The frequency of disease-modifying therapy usage in the group on statin treatment (51% interferon-beta 1a, 7% glatiramer acetate, 20% natalizumab, 9% no current disease-modifying therapy, with the remainder on combination therapies or chemotherapies) was similar to the group not receiving statins (43% interferon-beta 1a, 16% glatiramer acetate, 20% natalizumab, 8% no therapy, with the remainder on combination therapies or chemotherapies). There was no evidence for significant differences in the lipid profile variables among the interferon-beta, glatiramer acetate, natalizumab, combination therapy or chemotherapies and no current disease-modifying therapy groups (one-way ANOVA).

### Associations with Disability and Disability Changes

Higher total cholesterol to HDL ratio showed an association trend with baseline MSSS (Slope = 0.161 ± 0.092, Partial correlation coefficient *r_p _*= 0.080, *p *= 0.080) and with higher probability of occurrence of baseline EDSS ≥ 4.0 (*p *= 0.082, OR = 1.17). There was no evidence for associations for the other lipid profile variables or BMI. In the subset without statin treatment, the probability of occurrence of baseline EDSS ≥ 4.0 exhibited increasing trends with higher total cholesterol (*p *= 0.040) and cholesterol to HDL ratio (*p *= 0.017). There was no evidence for an association with HDL. Baseline MSSS trended higher with higher total cholesterol to HDL ratio (Slope = 0.23 ± 0.11, *r_p _*= 0.11, *p *= 0.038).

The associations of lipid profile variables with EDSS and MSSS changes are summarized in Table 3. Worsening EDSS changes were associated with higher LDL (*p *= 0.006), triglycerides (*p *= 0.025), total cholesterol (*p *= 0.001) and exhibited a trend with total cholesterol to HDL ratio (*p *= 0.047) levels. The EDSS change was not associated with higher HDL (*p *= 0.79). Similarly, worsening MSSS changes were associated with higher total cholesterol levels (*p *= 0.008); trends were also found with higher LDL (*p *= 0.012) and triglyceride (*p *= 0.037) levels. BMI was not associated with disability changes on either the EDSS or MSSS (results not shown). Qualitatively, similar results were obtained in the subset of patients who were not on statin treatment (results not shown).

**Table 3:** Lipid profile associations with disability changes.

		EDSS Change			MSSS Change		
		
Lipid Profile	Group	Slope ± SE	*r_p_*	*p-*value	Slope ± SE	*r_p_*	*p-*value
HDL	All	0.001 ± 0.003	0.012	0.79	0.000 ± 0.005	-0.010	0.83
	No statin	0.000 ± 0.004	-0.008	0.87	-0.003 ± 0.005	-0.030	0.56

LDL	All	0.004 ± 0.002	0.13	0.006	0.005 ± 0.002	0.12	0.012
	No statin	0.003 ± 0.002	0.093	0.078	0.006 ± 0.003	0.11	0.038

Total cholesterol	All	0.004 ± 0.001	0.15	0.001	0.005 ± 0.002	0.12	0.008
	No statin	0.004 ± 0.002	0.12	0.020	0.005 ± 0.002	0.11	0.030

Triglycerides	All	0.001 ± 0.0006	0.10	0.025	0.002 ± 0.0009	0.096	0.037
	No statin	0.002 ± 0.007	0.12	0.025	0.002 ± 0.001	0.10	0.055

Cholesterol to HDL ratio	All	0.083 ± 0.042	0.091	0.047	0.079 ± 0.062	0.059	0.20
	No statin	0.093 ± 0.050	0.098	0.062	0.12 ± 0.074	0.082	0.12

Significant *p*-values are underlined.

SE is standard error of the slope and *r_p _*is the partial correlation.

These results indicate that LDL, triglyceride and total cholesterol lipid profile variables are associated with disability changes in MS patients.

### Associations with MRI

Higher HDL levels were associated with a lower probability for the presence of CEL (*p *= 0.01) and lower CE-LV (*p *< 0.001). A qualitatively similar pattern of protective associations for higher HDL was found in the group not receiving statin treatment for the presence of CEL (*p *= 0.029, a trend) and for CE-LV (*p *< 0.001).

In contrast, higher triglyceride levels were associated with trends for a higher probability for the presence of CEL (*p *= 0.038) and with higher CE-LV (*p *= 0.023). There were similar trends for triglyceride levels with the presence of CEL (*p *= 0.060) in the group not receiving statins.

There was no evidence for associations between the presence of CEL and LDL (*p *= 0.80) or total cholesterol (*p *= 0.44) levels. There was also no evidence for associations between CE-LV with total cholesterol levels (*p *= 0.20). Greater levels of total cholesterol were associated as a trend with lower CEL number (*p *= 0.046) in part as a consequence of the HDL associations with CEL number. Lower CE-LV was also associated as a trend with lower levels of cholesterol to HDL ratio (*p *= 0.025). There was no evidence for associations of LDL with CEL number (*p *= 0.44) or CE-LV (*p *= 0.89) in patients not on statins.

There were no significant associations of T2-LV and T1-LV with any of the lipid profile variables (HDL, LDL, Triglycerides, total cholesterol and cholesterol to HDL ratio) or BMI. However, lower BPF values were associated with high total cholesterol levels (*r_p _*= -0.16, *p *= 0.033). There was also a trend toward an association between lower BPF values with higher total cholesterol in the sub-group that was not on statin treatment (*r_p _*= -0.16, *p *= 0.054).

## Discussion

In this paper, we have reported results indicating that lipid profile variables such as increased LDL, triglycerides and total cholesterol levels are associated with increased disability progression in MS. Higher HDL levels and lower levels of triglycerides were associated with decreased CEL activity whereas higher total cholesterol levels were associated with lower BPF.

The recruitment and extravasation of immune cells across the activated vascular endothelium of the blood brain is considered to a critical step in MS pathogenesis [1]. MS is also associated with significant amounts of cerebral vascular endothelial dysfunction [28,29] and with cerebral hypoperfusion [30,31]. Our working hypothesis is that the pro-inflammatory and thrombogenic processes associated with dyslipidemia could plausibly contribute to disease progression in MS via diverse mechanisms at the blood brain barrier vascular endothelium, e.g., by enhancing leukocyte recruitment, increasing endothelial dysfunction and by increasing the risk of hypoperfusion.

The effects size contributions of individual lipid profile variables to disability change were modest but significant: the partial correlation coefficient *r_p _*values were in the 0.10 - 0.15 range. We found greater EDSS worsening in patients with higher cholesterol (*p *= 0.001) and LDL (*p *= 0.006) levels at baseline. Similar associations were seen for MSSS, a disability measure with better metric properties that corrects the EDSS for disease duration. Nonetheless, our results provide mechanistic support, albeit indirect to the epidemiological findings of Marrie et al. who found that vascular comorbidities are associated with a substantially increased risk of disability progression in MS [22]. Long-term adherence to a low saturated fat diet has been implicated in better clinical outcomes in MS [32]. Although the MS cases in the Nurse Health Study cohort did not indicate associations between diet and the risk of developing MS, an association between obesity during adolescence has been reported [33].

The primary limitations of our study stem from its retrospective study design. Another caveat is the inclusion of statin-treated patients (22.2% of sample). Because hypercholesterolemia occurs with greater frequency in older male patients, the inclusion of the statin-treated sub-group introduces demographic heterogeneity. We did not find evidence for differences in overall lipid profiles in the statin-treated subset but the group on statin treatment was more frequently male, had greater mean age, disease duration, BMI, baseline EDSS scores and also a somewhat higher proportion of progressive MS, all of which would also be expected in an older and male MS patient group. This cluster of demographic characteristics is generally representative of statin treated patients in the population. All of our statistical analyses were corrected for age and sex to address demographic differences. In addition to their direct effects on cholesterol production, statins exhibit pleiotropic immunomodulatory effects *in vitro *[34] and in chronic and relapsing experimental autoimmune encephalomyelitis, an animal model of MS [35]. Cholesterol is a major component of myelin and statins may hinder remyelination by inhibiting cholesterol synthesis in the brain [36,37]. The studies of statin treatment in MS have likewise also yielded mixed results [38-42]. Therefore, to further address limitations imposed by the pleiotropic effects of statins and the representative demographic differences, we conducted sub-analyses in patients who were not on statin therapy. Our statin treated group did show a lower CEL number and CE-LV, with a higher T1-LV and a trend toward decreased BPF compared to the non-statin group. We avoided comparing the groups with and without statin treatment in results because this study was not designed to address the specific role if any of statins in MS therapeutics.

In a study of 30 MS patients, statin treatment resulted in a significant decrease in the number and volume of CEL on serial monthly MRI [39]. A post hoc analysis of the interferon-beta treated control arm of the SENTINEL study did not indicate an effect of statins on adjusted annualized relapse rate, disability progression, number of CEL, or number of new or enlarging T2-hyperintense lesions over 2 years [40]. The STAYCIS trial to assess statin treatment in slowing the conversion of CIS did not meet its primary endpoint [41]. The SIMCOMBIN trial indicated that statin treatment did not provide benefit in MS patients on interferon-beta [43].

Our data suggest a negative influence of high cholesterol and triglycerides on disease course and a favorable influence of higher HDL levels on acute inflammatory activity in MS patients. Lifestyle changes including adoption of a healthier diet and regular exercise in order to improve the serum lipid profile may be beneficial for MS patients to improve their neurological condition.

## Competing interests

Dr. Weinstock-Guttman received honoraria for speaking from Teva Neuroscience, Biogen Idec and EMD Serono. She also received financial support for research activities from National Institute of Health, National Multiple Sclerosis Society, National Science Foundation, Department of Defense, EMD Serono, Accorda, Biogen Idec, Teva Neuroscience, Cyberonics and the Jog for the Jake Foundation.

Murali Ramanathan received research funding from EMD Serono, Pfizer, Novartis, the National Multiple Sclerosis Society, the Department of Defense, National Institutes of Health and National Science Foundation. He received compensation as a consultant for Netezza, BiogenIdec and Allergan and for serving as an Associate Editor from the American Association of Pharmaceutical Scientists. These are unrelated to the research presented in this report.

Dr. Zivadinov has received speaker honoraria and consultant fees from Teva Neurosciences, Biogen Idec, Questcor, Genzyme and EMD Serono; and received research support from the National Multiple Sclerosis Society, the Biogen Idec, Teva Neuroscience, Teva Pharmaceuticals, Genzyme, Questcor, Bracco and Greatbatch.

Bijal Mehta, received honoraria for speaking from Biogen Idec.

Naeem Mahfooz, Ellen Carl, Allison Drake, Jaclyn Locke, Barbara Teter, Sara Hussein, Jacqueline Durfee, and Niels Bergsland do not have any conflicts of interest.

## Authors' contributions

BWG contributed to study design, oversaw all clinical aspects of the project including clinical data acquisition, data analysis and interpretation and manuscript preparation. RZ contributed to study design, MRI data acquisition, data interpretation and manuscript preparation. EC contributed to MRI data acquisition. AD contributed to clinical data acquisition. JS contributed to clinical data acquisition. BT oversaw clinical data acquisition. SH contributed to data acquisition. BM contributed to clinical data acquisition. MW contributed to clinical data acquisition. JD contributed to MRI data acquisition. NB contributed to MRI data acquisition. MR contributed to study design, data analysis and interpretation and manuscript preparation. Al authors read and approved the final manuscript.

## Supplementary Material

Additional file 1 **Additional file **1**contains MRI Acquisition Protocol, Image Analysis methods and Table S1**. Click here for file
