# Supplementary material for: Serum lipid profiles are associated with disability and MRI outcomes in multiple sclerosis
Source: J Neuroinflammation. 2011 Oct 4;8:127. doi: 10.1186/1742-2094-8-127 (PMC3228782; doi:10.1186/1742-2094-8-127)
Supplement: Additional file 1 — contains MRI Acquisition Protocol, Image Analysis methods and Table S1. [file 1742-2094-8-127-S1.DOC]

**ADDITIONAL FILE 1**

**MRI Acquisition Protocol**

Quantitative MRI analysis was available for 210 of 492 patients at baseline. Patients underwent brain MRI on a 1.5T GE Signa Excite HD 12.0 Echospeed 8-channel scanner (General Electric, Milwaukee, WI) within 90 days from the blood draw. MRI sequences included multi-planar dual fast spin-echo (FSE) proton density (PD) and T2-weighted image (WI), Fluid-Attenuated Inversion-Recovery (FLAIR), spin echo (SE) T1-WI with and without contrast, and 3-dimensional spoiled gradient echo (3D-SPGR). Pulse sequence characteristics for 1.5 Tesla MRI were as follows: the 3D-SPGR T1W was acquired with a 256 x 256 matrix and a FOV of 25.6 cm, for an in-plane resolution of 1 x 1 mm^2^; the PD/T2, FLAIR and SE T1 sequences were acquired with a 256 x 192 matrix and FOV of 24 cm, for an in-plane resolution of 0.94 x 1.25 mm^2^. Other parameters were: for the PD/T2 sequence, TE1/TE2/TR = 10/90/7475 ms, ETL = 12, 46 slices, 3 mm thick, no gap, one average, AT=4:07; for 3D-SPGR T1W, Flip Angle = 20, TE/TR = 6/27 ms, 110 slices, 1.5 mm thick, no gap, one average, AT=9:55; for SE T1 images acquired before and 5 min after contrast administration (using a single dose intravenous bolus of 0.1 mMol/Kg Gd-DTPA), TE/TR=12/450 ms, 46 slices, each 3 mm thick with no gap, two averages, AT=6:40; and T2 FLAIR scans, TE/TI/TR=120/2000/8000 ms, 46 slices, 3 mm thick, no gap, one average, AT=5:21.

**Image Analysis**

Image analysis was performed in the Buffalo Neuroimaging Analysis Center, Buffalo, NY. The MRI analysts were blinded to patients’ clinical and lipid characteristics. Lesion Measures: The T2-, T1- and contrast enhancing (CE) lesion volumes (LVs) were measured using a semi-automated edge detection contouring/thresholding technique previously described(1). CE lesion number was also obtained. For brain extraction and tissue segmentation, the SIENAX cross-sectional software tool was used (version 2.6), with corrections for T1-hypointensity misclassification using an in-house developed in-painting program (2). Brain parenchymal fraction (BPF) was calculated to correct for head size (2).

**Additional Table 1.** Demographics, clinical characteristics and lipid profiles of the subsets with and without MRI available.

| **Variable** | **With MRI** | **Without MRI** | ***p*-value** |
| --- | --- | --- | --- |
| Females: Males (% Female) | 159: 51 (75.7%) | 211: 71 (74.8%) | 0.83^§^ |
| MS course:  Relapsing-remitting  Secondary progressive  Primary progressive | 182 (86.7%)  22 (10.5%)  6 (2.9%) | 213 (75.5%)  60 (21.2%)  9 (3.2%) | 0.002^‡^ |
| Age*, years | 46.4 ± 10.6 | 47.6 ± 10.9 | 0.21 |
| Disease duration*, years | 12.1 ± 10.0 | 13.4 ± 10.2 | 0.18 |
| Median EDSS* (IQR) | 2.50 (2.0) | 2.50 (3.0) | 0.24^#^ |
| MSSS | 3.74 ± 2.4 | 3.83 ± 2.5 | 0.69 |
| Time to follow-up, years | 1.99 ± 0.98 | 2.3 ± 1.0 | 0.002 |
| Statin usage | 19.1% | 24.5% | 0.19^§^ |
| Body mass index, kg/m^2^ | 27.6 ± 6.3 | 27.9 ± 6.6 | 0.63^¶^ |
| HDL, mg/dL | 55.5 ± 17.1 | 54.9 ± 16.2 | 0.71^¶^ |
| LDL, mg/dL | 117 ± 33.2 | 115 ± 32.5 | 0.63^¶^ |
| Total cholesterol, mg/dL | 198 ± 37.7 | 197 ± 38.5 | 0.83^¶^ |
| Triglycerides, mg/dL | 129 ± 83.5 | 135 ± 81.8 | 0.40^¶^ |
| Cholesterol to HDL ratio | 3.86 ± 1.46 | 3.84 ± 1.19 | 0.87^¶^ |

* At time of baseline lipid profile assessment.

^§^ Fisher exact test.

^‡^ Fisher exact test for presence of secondary progressive or progressive forms of MS.

# Mann-Whitney test

^¶^ *p*-values for MRI available variable from regression analyses with sex, disease duration, statin use and MRI available as predictor variables.

**REFERENCES**

1. Zivadinov R, Rudick RA, De Masi R, et al. Effects of IV methylprednisolone on brain atrophy in relapsing-remitting MS. Neurology 2001;57:1239-1247.

2. Zivadinov R, Weinstock-Guttman B, Hashmi K, et al. Smoking is associated with increased lesion volumes and brain atrophy in multiple sclerosis. Neurology 2009;73:504-510.
